# Monthly Report of Diseases

**Published:** 1800-07

**Authors:** 


### MONTHLY REPORT of DISEASES,

Admitted under the Care of the Physicians of the Finsbury Dispensary, St. John's Square, Clerkenwell.

*The District, in which the Patients of the Finsbury Dispensary are visited, comprehends the Parishes of St. James, and of St. John, Clerkenwell; of St. Luke; of St. Sepulchre within and without; of St. Bartholomew, the Great and the Less; the Liberties of the Rolls, and of Glass-House Yard; the Town of Islington; the Parishes of St. Pancras; of St. Andrew, Holborn; and of St George the Martyr, Queen's-square. This Tract of Ground may properly enough be termed, a North Western District of the Metropolis.*

List of Diseases, &c. from May 20, to June 20.

| | No. of Cases. | | No. of Cases. |
|---|---|---|---|
| Hæmorrhois | 4 | Sore Throat | 1 |
| Worms | 7 | Continued Fever | 10 |
| Prurigo | 11 | Pneumonia | 2 |
| Schrophula | 3 | Dysentery | 3 |
| Jaundice | 2 | Amenorrhœa | 13 |
| Paralysis | 3 | Leucorrhœa | 9 |
| Cough and Dyspnœa | 14 | Asthenia | 10 |
| Dyspepsis | 27 | Phthisis | 12 |
| Chronic Rheumatism | 12 | Hysteria | 6 |
| Acute Rheumatism | 2 | Dropsy | 7 |
| Lumbago | 4 | Cephalæa | 5 |
| Menorrhagia | 10 | Infantile Diseases | 18 |
| Diarrhœa | 6 | Mania | 3 |
| Hæmoptysis | 6 | | |

It

It appears from the above Report, that the diseases which have principally prevailed during the last month are *dyspeptic*, or those that originate from some fault in the organs of digestion. This is, in a certain degree, to be accounted for by the alteration that has taken place in the temperature of the air, which, whilst it has proved more favourable to the relief of catarrhal and rheumatic affections, may, by its increased warmth, have a tendency to relax the tone of the stomach, and thus predispose to those morbid symptoms which are more peculiarly connected with that viscus.

The disorders of the stomach which have occurred, might, in a large proportion of cases, be clearly traced in men, to the abuse of spirituous liquors; and in women, in addition to the abuse of spirituous liquors, to which, after they have once passed over the bounds of sobriety, they are, more than the other sex, completely and irretrievably devoted, an intemperance in the use of tea. Although this, in a *moral* view, is surely far less dangerous than inebriating stimuli, is not, perhaps, much less so in its physical effects upon the constitution. Besides, that from the degree of debility and depression which it induces, a person is naturally led to have recourse to what are called *tonic* medicines, or to exhilirating cordials.

A remarkable case has occurred of an idiotic girl, about fifteen years of age, who is uniformly thrown into a state of the most violent madness by the hearing of any kind of instrumental music, such as the ringing of bells, the notes of an organ, &c. After the hearing these or any other similar instrument, she, for hours, endeavours to imitate the sound with her voice. What makes this case still more singular, is, that according to her mother's account, she is not in any degree affected in the same way by *singing*.

There was another case of mania, which the sister of the patient attributed to the influence of religious enthusiasm. But whether, in this particular instance, the religion gave rise to the insanity, or the insanity to the religion, it was not in the power of the medical attendant to ascertain.

One of the instances of cephalæa, in the above list, was remarkable from its alternating with an ulceration of the breast. Whilst the latter, either by time, or the application of surgical skill, gradually got better, the former as gradually came on. This alternation had taken place in repeated instances. The pain in the head was at its greatest violence at the period of the patient's first attendance at the Dispensary, when the sore was completely healed. As a substitute for the discharge thus stopped, an issue was ordered in the arm, in consequence of which, and partly perhaps in consequence of the Peruvian bark, which which was alſo preſcribed, and the enjoyment of country air, the patient for ſome time paſt has not, in any conſiderable degree, ſuffered from either of the complaints with which ſhe had been before afflicted.

In the caſes of amenorrhoea that have occurred during the period that the drawer up of this report has attended the diſpenſary, the preparations of ſteel have exhibited a decided ſuperiority over thoſe of the Peruvian bark, or indeed, any other remedy taken into the ſtomach.

Electricity in one inſtance proved almoſt immediately ſucceſsful. It is to caſes like theſe, where a *ſudden agitation* of the ſyſtem is required, that this ſtimulus ſeems peculiarly adapted. In a caſe of paralyſis in which it was adminiſtered, it produced ſome appearance of temporary relief, but the patient died on the ſucceeding day. Perhaps it may be laid down as a general rule, that *chronic* diſeaſes are not to be cured by *violent ſtimuli*, but rather by the application of thoſe gentle remedies that gradually reſtore the ſtrength of the conſtitution.

A caſe equally remarkable and melancholy, has remained for a very long period under the care of the diſpenſary. It is that of a young woman who, for many years paſt, has been confined to her bed in a ſtate of univerſal ſpaſm. She lies rigid and motionleſs, with her eyes more than half cloſed, and every other organ of ſenſe almoſt completely ſhut againſt external impreſſion. The phyſician who attended her, by ſpeaking in her ear as loud as it was poſſible for him to do, ſucceeded only ſo far as to produce a motion of the lips that betrayed an ineffectual endeavour for utterance. It ſeems to be a caſe in which there is an habitual abſence of actual ſenſation, although, by ſome violently exciting cauſe, the ſenſibility may, at times, be imperfectly awakened. Lying in ſuch a ſtate, with ſcarcely any ſymptom of vitality but a feeble reſpiration, ſhe can be regarded as little elſe than a *breathing corpſe*. Upon this curious caſe being ſtated to a perſon of diſtinguiſhed learning and acuteneſs, he ſuggeſted it as a poſſibility, that conſciouſneſs might ſtill *exiſt*, although it was unable to *appear*, in conſequence of the voluntary muſcles uſually employed in expreſſing it, refuſing in this inſtance to diſcharge their accuſtomed office. This, it is to be hoped, is not actually the caſe. Nothing is more terrific to the imagination than the idea of being buried alive; and what mode of being buried alive can be conceived more truly horrible than for the *ſoul to be entombed in the body!*

J. R.
W. W.

*Hatton Garden.*

